# Epidemiologic research on lung damage caused by humidifier disinfectants

**DOI:** 10.4178/epih.e2016031

**Published:** 2016-07-20

**Authors:** Moo-Song Lee, Hwa Jung Kim

**Affiliations:** 1 Department of Clinical Epidemiology and Biostatistics, Asan Medical Center, University of Ulsan College of Medicine, Seoul, Korea; 2 Department of Preventive Medicine, University of Ulsan College of Medicine, Seoul, Korea

**Keywords:** Humidifiers, Disinfectants, Lung disease, Republic of Korea, Case-control studies, Disease outbreaks

## Abstract

In April 2011 a tertiary hospital located in Seoul, Korea reported several cases of severe respiratory distress of unknown origin in young adults. To find the route of transmission, causative agent and patient risk factors of the outbreak, an investigation of the epidemic was initiated. A hospital based case-control study was conducted to indicate that humidifier detergent use was the cause of the outbreak. This information led the Ministry of Health and Welfare of Korea issued an order that humidifier detergents should be withdrawn from the market. Here, we describe the major events of planning, execution, and interpretation of the study, and discussions between researchers and public authorities following the decision to perform an epidemiologic study, chronologically.

## INTRODUCTION

In late April 2011, six perinatal female patients were admitted to the respiratory intensive care unit of a general hospital (hereafter A-hospital) in Seoul, Korea for a lung disease with unknown cause [1,2]. The disease began with slight respiratory symptoms, and progressed to respiratory failure unresponsive to any treatment [1]. The medical staff requested an epidemiologic study on this unreported new disease from the Korea Centers for Disease Control and Prevention (KCDC). Officials of the National Epidemiology Center of the KCDC visited A-hospital to investigate. An “Emergency committee meeting for severe respiratory disease of perinatal mothers” was held on May 6, in which KCDC officials, KCDC committee members, and professors in the departments of pulmonology, infectious disease, pathology, radiology, pediatrics, and preventive medicine in A-hospital participated, to discuss emergence of a novel disease, epidemic potential, and urgency of epidemiologic study.

In the emergency committee meeting, disease states, prognosis, and various test results of the six patients reported by A-hospital were reviewed, and epidemic potential, possible etiology, and future planning were discussed. Considering the unusual clinical, radiologic, and pathological findings, and lack of response to treatments for other lung diseases, it was determined to be a novel disease. On the other hand, similar findings were observed in lung tissues of adult males who lived with the patients, and it was reported that a lung disease with unknown cause has also been found in children since 2006. Various causes were suggested, including novel viral infection, yellow dust, drugs, and humidifier use.

On May 7, the KCDC requested an epidemiologic study of A-hospital, with a research team composed of clinical and preventive medicine staff in A-hospital, outside specialists, and KCDC officials (epidemiologic investigators). The purpose of the epidemiologic study was to objectively define the disease with unknown cause, investigate the extent of occurrence, and determine its etiology, in order to prevent involvement of more patients.

After three months of epidemiologic study [2] and cytotoxicity testing, the KCDC officially announced on August 31, 2011 that humidifier disinfectants (HDs) should be banned from use, and ordered removal of HDs from the market on November 11, based on animal inhalation toxicity test results.

During the six months from May to November 2011, the etiology of a new disease was revealed based on epidemiologic study results and biological evidence, including animal tests. This report will describe the epidemiologic study of diseases caused by HDs that the author performed as principal investigator (May to July 2011), including the chronology, characteristics, lessons, and limitations of the study.

## CHRONOLOGICAL FLOW OF THE EPIDEMIOLOGIC STUDY OF LUNG DISEASE WITH UNKNOWN CAUSE

This section chronologically describes the major events of planning, execution, and interpretation of the study, and discussions between researchers and public authorities following the decision to perform an epidemiologic study, from May 7, 2011 (officially decided in late May) to August 31, 2011, with the official announcement that HD was the cause (Tables 1 and 2).

- May 7: Based on the emergency committee meeting on May 6, an epidemiologic study was planned. At that time, the target disease was severe pneumonia with unknown cause during pregnancy.
- May 12: According to the diagnostic criteria, perinatal female patients in A-hospital and suspected patients in other hospitals were identified. Since similar diseases were identified in family members of patients, adult males were also included in the study.
- May 13: Among nine patients in A-hospital, two were deceased, two underwent lung transplantation, and seven were perinatal females. Additional nine patients were identified in other hospitals. Disease onset was from February to early May, with no subsequent reports of new patients [1]. Pathologic findings indicated lung damage by inhalation, and potential common underlying diseases and autoimmune disorders were excluded.
- May 15: Respiratory specimens from patients were tested to result in exclusion of viral infections from potential causes.
- May 16: Based on clinical data including findings from chest radiographs, operational criteria (working definition) for the disease were established [1,2]. However, study expansion outside of A-hospital was unpalatable due to the Personal Data Protection Act of Korea, and approval from the institutional review board (IRB) of each medical institution.
- May 17: The electronic medical records of A-hospital were searched with keywords such as adult females, ground glass opacities (GGO), and diffuse and disseminated, to select suspected patients. Most pathological findings in lung tissue specimens indicated fibrosis rather than inflammation; so that the disease was expected to be caused by inhalation of an unidentified substance.
- May 18: Target disease of investigation were determined to be a new disease, but similar pediatric cases which were previously reported as acute interstitial pneumonitis in 2006 were found [3]. However, initial epidemiologic study was decided to be conducted among adults only for efficacy; potential cases were selected firstly by chest radiographs, and then case confirmation were made through medical records review by clinicians. Humidifier or HD were suggested as possible cause.
- May 19: A nationwide epidemiologic study was planned and, potential patients were to be identified through the Respiratory Failure Society, the Society of Thoracic Radiology, and the Pediatric Allergy and Respiratory Disease researcher network. Analysis of HDs were suggested.
- May 24: During 2011, 11 patients were found at A-hospital.
- May 25: Questionnaire items considering various etiology were discussed, and were decided to be applied to both patients and cohabitating families. It was found that HDs were not in the market after May, because they were seasonal products.
- May 26: The IRB of A-hospital waived the review for study of patient groups. Three types of HDs were sent to a related agency to evaluate then contents.
- May 27: Face-to-face interviews were started.
- May 30: Respiratory disease patients and peripartum females were selected to be the control group.
- June 1: Study progress was reported in the second KCDC committee meeting. Difficulty to conduct this investigation outside of A-hospital were discussed.
- June 2: Potential and confirmed cases with disease onset before 2011 were identified, so that the target disease was evaluated to be occurred prior to 2011. Chemical components of HDs were identified, and design of animal test was discussed.
- June 3: The control group were decided to be select four inpatients at A-hospital per each patient, matched for sex, age, region, and time of hospitalization.
- June 7: It was decided to initiate a case-control study in A-hospital first, to be expanded to other hospitals later.
- June 8: Telephone surveys were initiated with patients whose onset was prior to 2011. Chances of fungal infection were discussed.
- June 10: Living environments were evaluated by visiting some patient houses. Material Safety Data Sheets for HDs were identified.
- June 13: House visit were decided to target all patients for in-depth interviews.
- June 14: A total of 32 suspected patients after 2001 were identified in A-hospital according to the operational criteria.
- June 17: An agency to perform animal inhalation toxicity testing for HDs was decided.
- June 22: Following approval by the IRB of A-hospital, a control group survey study was initiated. Some HD products of Cefu and HD products from Oxi, Lotte Mart, and Homeplus were found to contain oligo(2-(2-ethoxy)ethoxyethyl guanidium chloride and polyhexamethylene guanidine, respectively.
- June 23: A progress report meeting was held. Among 32 suspicious patients identified in A-hospital. and 28 patients were confirmed (18 were cases of 2011, and other 10 were from 2006 to 2010) and 18 patients completed the survey. Of 15 females, 14 had used HDs, as did all three males. It was decided to select a control group comprising two patients with respiratory disease and two postpartum females per each female patient; four male patients with respiratory disease were also selected [2].
- June 24: Constructed questionnaire was finalized for the survey among controls.
- June 28: A committee meeting on “unknown cause lung damage” was held.
- June 29: The face-to-face survey for control group with respiratory disease and peripartum women was completed.
- June 30: Viral test results identified no suspected cause. Patients with allergy disease were diced to be added for control group.
- July 1: It was decided to add investigation of the components of HDs and animal testing to the epidemiologic study.
- July 4: Houses of familial cluster cases were visited, to collect samples of their living environments.
- July 7: Elevated odds ratio (OR) of HDs, humidifiers, and fungi were observed [2].
- July 8: The Korea Institute of Toxicology was identified as the animal inhalation toxicity testing agency for HDs.
- July 11: Additional surveys which were conducted among additional control group were completed. Due to the low feasibility of expanding participants outside of A-hospital, the case-control study was decided to be completed.
- July 12: Study results - that HDs were considered as the epidemiologic cause - were reported to the KCDC and Ministry of Health and Welfare. It was necessary to perform an animal inhalation toxicity test, and to identify components of HDs in detail.
- July 13: It was decided to recruit additional local community control groups [4]. In addition, the necessity of supplementing biological evidence with animal test results was discussed.
- July 18: The final results were reported to the Ministry of Health and Welfare, KCDC, and the advisory committee of the epidemiologic study including 18 cases and 121 controls. Crude and adjusted (for sex, age, and fungal exposure) OR of HDs were 47.3, 48.8 respectively (Table 3) [2]. It was decided to further perform pneumonocyte and animal inhalation toxicity testing for HDs.
- July 20: It was decided to initiate a study on pediatric patients starting in August. As HDs were indicated for cause of the disease, in-depth interview and home visits were scheduled to begin July 21.
- July 25: It was decided to add questions to assess dose-response relationships for HD to the existing questionnaire, and to conduct a survey on a local community control group [4]. It was also decided that the KCDC would perform all epidemiologic studies in children [3,5], and all cell and animal studies. For epidemiologic study in children, the team at A-hospital joined the research team, and used a similar study design [3,5].
- August 1: Additional questions asked during the in-depth interviews were added for local community control groups questionnaires [4]. To obtain biological evidence for causality, it was decided to perform cytotoxicity and animal testing with intratracheal instillation, with results to be reported in August.
- August 8: In-depth and home visit study of cases were terminated, and a total of 15 patients were evaluated. As a preliminary step for animal inhalation toxicity testing (to determine particle sizes emitted in the air), a HD generation test was conducted.
- August 11: Progress of cytotoxicity testing was reported, the 50% lethal concentration was calculated, and pneumonocyte apoptosis was observed.
- August 18: Particle emission test results by the Korea Institute of Toxicology showed that HDs were able to invade even terminal bronchioles, and tests confirmed cytotoxicity of HDs. In addition, animal testing with exposure to HD by intratracheal instillation resulted in weight loss and histopathologic inflammation, which confirmed toxicity in animals.
- August 30: Results of epidemiologic study, cell experiments, and particle emission tests were discussed in the advisory committee meeting composed of experts in “lung damage with unknown cause” (attendees included members of the epidemiologic study advisory committee; members of the expert committee in infectious disease, environmental health, toxicity, and pulmonology; the contracted research team; and KCDC officials).
- August 31: A press conference was held to announce that HDs were suspected as a risk factor for lung damage with unknown cause, and avoidance of their use and release was recommended until the causal relationship was finally confirmed.
- September 2: A preparative meeting for study on children was held.
- September 22: The research contract were made to perform a nationwide investigation on cases of severe lung disease with unknown cause in 2011.
- November 10: The second expert epidemiologic study committee meeting was held. Pathologic findings of mice exposed to HD were identical to those in lung disease patients with unknown cause.
- November 11: The Ministry of Health and Welfare announced that HDs were the cause of the lung damage with unknown cause, and ordered the removal of six types of disinfectants.
- From 2012 to date: No additional case of lung damage in adults and children due to HD has been reported. In addition, the case-control study on interstitial pneumonia in children was performed in the same way as the present case-control study, and also revealed HDs as the cause [3,5].

## ANALYSIS OF THE EPIDEMIOLOGIC STUDY ON HUMIDIFIER DISINFECTANTS

The present study is rare for having identified a new cause of lung damage due to HD. Several study characteristics, limitations, and lessons should be noted.

First, this was a multidisciplinary study of a disease epidemic reported by A-hospital, and performed by a collaboration between the hospital, a research team of the school of medicine (over 20 individuals from the departments of pulmonology, radiology, pathology, pediatrics, and preventive medicine), epidemiologic investigators of the KCDC epidemiology division, and over 20 experts from outside universities and institutes.

Second, the cause of a nationwide epidemic was only revealed through data from A-hospital. Because of the lack of clinical consensus for an epidemic of new disease, difficulty with medical data collection due to the Personal Data Protection Act of Korea, and the absence of a rapid institutional review process for a public study, the epidemiologic study was performed through a single agency.

Third, electronic medical records were actively utilized to determine if this was a new disease, to estimate the epidemic scale, and to define operational criteria for the disease. In the process of summarizing early reported clinical, radiologic, and pathological findings, the present epidemiologic study used common keywords, such as GGO, diffuse, and disseminated. The keywords were applied to electronic hospital medical records; potential patients were rapidly selected and evaluated by medical staff and radiologists to identify corresponding patients.

Fourth, after epidemiologic identification of HDs as a risk factor, experimental studies were rapidly performed to prove biological relevance. Since HDs are manufactured on a large scale, experimental evidence showing biological probability was required; epidemiologic evidence such as size of cross ratio and dose-response relationships was also required. For determination of exposure environment and disinfectant concentration in animal inhalation toxicity testing, toxicology, epidemiology, and clinical research teams established a feasible plan, which resulted in objective evidence of toxicity.

Fifth, an epidemiologic study should be performed using methods based on clinical knowledge. Thus, it is necessary for clinicians and epidemiologists to cooperate closely in all decision-making and research. A lack of communication might result in different data sets and even different conclusions. Clinicians and epidemiologists of A-hospital performed the study using data of A A-hospital alone (since a multi-agency study was impossible); however, this seemed to result in an uncomplicated and successful study.

## CONCLUSION

The present epidemic investigation shows that the use of home humidifier detergent could lead to a new lung injury. As lots of household chemicals are in use, and may pose an unanticipated hazard to human health. Proper system for safety monitoring among unrevealed household chemicals should be conducted.

## Figures and Tables

**Table 1.** Role of each participants of the epidemiologic investigation

	Asan Medical Center/University of Ulsan School of Medicine	Government agency and institute	Other academic experts and research societies
Stage 1 (hospital-based epidemiologic study)	Recognition of case by ICU medical staff and report - Departments of pulmonology and infectious disease - Departments of radiology and pathology	Reported to the Center for Infectious Disease Control in KCDC	Inquiry to the Respiratory Failure Society for similar patients
	Formation of epidemiologic study team in the hospital - Laboratory of preventive medicine and medical statistics department - Departments of pulmonology, infectious disease, pediatrics, and obstetrics/gynecology - Departments of radiology, pathology, and laboratory medicine - Medical information center and medical record center	Oversight support by the Center for Infectious Disease Control in KCDC - Division of epidemiology (epidemiologic investigator) Test support by the Division of Respiratory Viruses of the Korea National Institutes of Health	Identification of similar patients through the Respiratory Failure Society and the Society of Thoracic Radiology
Stage 2	Cooperation for epidemiologic study in the hospital	Animal test performed at the Department ofInhalation Research of the Korea Institute of Toxicology The Center for Infectious Disease Control in KCDC performed investigation of local community control group -Division of epidemiology (epidemiologic investigators, etc.)	Epidemiologist consultation Consultation by experts in environmental medicine and industrial medicine Toxicologists

ICU, intensive care unit; KCDC, Korea Centers for Disease Control and Prevention.

**Table 2.** Research steps of the epidemiologic investigation

	Date	Progress
Stage 1 (hospital-based epidemiologic study)		**Identification of epidemic and measurement of scale**
	7 May	Investigation of whether this was an epidemic; Since this was a severe lung disease of perinatal females that was not observed previously, it was considered to fit the definition of an epidemic
		Establishment of working (operational) definition
	8 May-15 May	Revision and complementation of operational criteria; Patients who showed similar clinical patterns other than perinatal females were identified, and common characteristics were derived by reviewing their radiologic, pathological, and clinical findings; Lung damage that showed ground glass opacities in radiologic findings and no response to existing treatments for lung disease defined the operational criteria
	16 May-2 Jun	Identification of patients according to operational criteria: Chest CT images of patients identified through search of electronic medical record were re-inspected, and patient group with corresponding clinical findings was identified; A total 28 patients were identified after 2011
		**Identification of epidemic characteristics through descriptive epidemiologic study**
	7 May-2 Jun	Various characteristics of identified patients group (28 patients in Asan Medical Center) were reviewed (Note) 5/26/2011: IRB decided to waive review of study on patient group - Temporal characteristics: clear seasonality, mostly found from early spring to early summer - Spatial characteristics: nationwide, no regional specificity - Demographic characteristics: mostly female patients aged in their 20s and 30s, relatively fewer male patients
		Establishment of hypothesis for epidemic cause - It was highly likely to be a disease caused by inhalation of toxic substance - In particular, considering that bronchiolar area was mostly invaded, it was suspected that the size of corresponding particles should be extremely small
		**Hypothesis testing through analytical epidemiologic study**
	2 Jun-29 Jun	Including materials with respiratory toxicity, case-control study was performed on known risk factors for respiratory disease (Note) 2/22/2011: IRB approval for the case-control study 28 patients were identified as patient group; control group included patients admitted for respiratory and allergic disease and patients admitted for delivery of babies Through survey with 18 patients in patient group and 121 patients in control group, humidifier disinfectants were found to be the epidemiologic cause
Stage 2		**Experimental study for biological probability**
	1 Jul-11 Aug	*In vitro* study: cytotoxicity test
	8 Jul-18 Aug	Humidifier disinfectant particle generation test
	8 Jul-10 Nov	*In vivo* study: inhalation toxicity test
		**Humidifier disinfectant-related public activity**
	31 Aug	Press conference to recommend avoidance of use and release of humidifier disinfectants
	11 Nov	Enforced collection of humidifier disinfectants ordered

CT, computed tomography; IRB, institutional review board.

**Table 3.** Relationship between exposure to HDs and lung damage in case-control study

	Adult patients^1^			Crude OR	Pediatric patients^2^			Crude OR
	Patient	Control	Subtotal		Patient	Control	Subtotal	
Hospital-based case-control study				47.3				∞
HD used	17 (94.4)	32 (26.4)	49		16 (100)	11 (23.4)	27	
HD not used	1 (5.6)	89 (73.6)	90		0 (0)	89 (73.6)	36	
Subtotal	18	121	139		16	47	63	

Values are presented as number (%).

HD, humidifier disinfectant; OR, odds ratio.

1 From Kim HJ, et al. Thorax 2014;69:703-708 [2].

2 From Yang HJ, et al. PLoS One 2013;8:e64430 [5].
