# Supplementary material for: Epidemiologic research on lung damage caused by humidifier disinfectants
Source: Epidemiol Health. 2016 Jul 20;38:e2016031. doi: 10.4178/epih.e2016031 (PMC5081306; doi:10.4178/epih.e2016031)
Supplement: Supplementary file 1 [file epih-38-e2016031-supplementary.pdf]

가습기 살균제 폐 손상 역학조사의 경험

울산의대 예방의학교실/서울아산병원 의학통계학과 이무송, 김화정

I. 서론

2011년 4월 하순 서울시내 종합병원(이하 'A병원'이라 함) 호흡기 중환자실에 주산기(perinatal) 여성 6명이 원인 미상의 폐 질환으로 입원하고 있었다. 1개월 전후의 가벼운 호흡기 증세로 시작해서 모든 치료에 반응하지 않는 호흡부전으로 진행하는 특징을 가진 특이한 질병으로 생각되었다. A병원 의료진은 이전에 보고된 바 없는 새로운 질병의 역학조사를 질병관리본부(이하 '질본'이라 함)에 요청하였다. 이후 질본 중앙 역학 조사반 실무자들이 병원을 방문하여 현황을 파악한 후 5월 6일 '산모 중증 호흡기질환 관련 긴급 자문회의'를 개최하였다. 질본 관계자 외에 질본 자문위원, A병원 호흡기내과, 감염내과, 병리학, 영상의학, 소아과 및 예방의학 교수가 참여하여 새로운 질병의 출현 및 유행 가능성, 역학조사의 시급성 등에 대해 논의하였다.

긴급 자문회의에서는 A병원에서 신고한 환자 6명의 질병 상태, 예후, 각종 검사결과 등을 검토하였으며, 유행(epidemic) 여부, 가능한 원인과 이후 계획 등의 안건을 논의하였다. 특이한 임상적, 영상의학적, 병리학적 소견을 보이는 점, 다른 폐질환의 치료에 반응하지 않는 점 등을 감안하여 새로운 질병으로 판단하였다. 한편 환자와 동거하는 성인 남자의 폐 조직에서 유사한 소견이 관찰되었고 2006

년 이후 소아에서도 원인 미상의 폐질환이 발생하였다는 보고가 있었다. 발병 원인에 대해서는 신종 바이러스 감염, 황사, 약물, 가습기 사용 등 다양한 가능성이 제기되었다.

질본에서는 5월 7일 A병원에 역학조사를 의뢰하였으며, A병원의 임상 및 예방의학 연구진, 외부 전문가 및 질본의 관계자(역학조사관 등)로 연구팀을 구성하였다. 역학조사의 목적은 원인 미상의 질병을 객관적으로 정의하여 발생 규모를 파악하고, 그 원인을 규명하여 더 이상의 환자 발생을 방지하는 것이었다.

3개월간의 역학조사 및 세포독성 시험을 거쳐 2011년 8월 31일 가습기 살균제 사용 자제를 포함한 질본의 공식 발표가 있었으며, 가습기 살균제의 동물 흡입 독성시험 결과에 따라 11월 11일 가습기 살균제의 공식 수거 명령이 내려졌다.

2011년 5월부터 11월까지의 6개월은 역학조사 결과와 동물시험 등의 생물학적 증거를 토대로 새로운 질병의 원인을 규명한 세계적으로도 드문 사례이다. 아래에서는 저자가 연구책임자로서 수행한 가습기 살균제 역학조사(2011년 5월부터 2011년 7월)의 과정을 시계열적으로 기술하고, 본 역학연구의 특징, 교훈과 한계

등을 정리하고자 한다.

Ⅱ. 원인 미상 폐질환 역학조사의 시계열적 흐름

아래에서는 5월 역학조사가 결정된 이후 연구의 기획, 수행, 해석 및 연구자-공공기관 간 협의과정에서의 주요 사건을 역학조사가 결정된(공식적인 결정은 5월 하순) 5월 7일 이후부터 가습기 살균제를 원인으로 추정하여 공식 발표된 8월 31일까지 시간 순으로 기술하였다.

2011년 5월 7일

5월 6일 A병원에서 개최된 긴급 자문회의를 토대로 역학조사를 수행하기로 하고, A병원/U의과대학 예방의학과 교수인 저자를 연구책임자로 결정하였다. 그 당시 대상 질병은 원인 미상의 중증 임산부 폐렴이었다.

2011년 5월 12일

잠정적 환자 진단기준에 따라 A병원의 임신 및 출산 직후 환자를 파악하고, 타 병원의 의심 환자를 확인하였다. 환자 가족 중 유사 질병이 확인되어, 성인 남자를 연구대상에 포함하였다.

2011년 5월 13일

5월 13일 현재 A병원 환자는 9명 중 2명은 사망하였고, 2명은 폐 이식을 받았으며, 9명 중 주산기 여성이 7명이었다. 또한 타 병원 환자 9명이 확인되었다. 질병은 2월부터 5월 초까지 발병하였으며, 새로운 환자 발생이 없는 상태였다. 병리 소견은 흡입에 의한 폐 손상을 시사하는 소견이었으며, 공통 기저 질환, 자가면역성 질환의 가능성은 배제되었다.

2011년 5월 15일

환자의 호흡기 검체를 대상으로 각종 호흡기 바이러스 및 한타 바이러스 검사를 시행하였다. 이후 바이러스 감염의 가능성은 배제되었다.

2011년 5월 16일

흉부 영상 판독소견을 포함한 임상 데이터를 정리하여 공통점을 도출하여, 질병의 실행적 정의(working definition, operational criteria)를 구축하였다. 그러나 본 질환이 기존에 존재하였던 질병인지, 이전에 보고되지 않은 새로운 질병인지에 대해서는 A병원 외 모든 병원에서 컨센서스가 얻어지지는 않은 상태였다. 또한 타 병원으로 역학조사 범위를 확대하기 위해서는 개인정보보호법 준수, 각 의

료기관 내 연구윤리위원회(Institutional Review Board, IRB) 승인이 필요하였다.

2011년 5월 17일

A병원 전자의무기록에서 성인 여자, 확산된 유리 음영(ground glass opacities(GGO), diffuse, disseminated) 등의 키워드로 검색한 결과, 의무기록을 통해 의심 환자를 선별하는 것이 가능하였다. 아이가 발병한 후 산모가 발생한 예도 1건 있었다. 또한 확보된 환자 폐 조직의 병리학적 소견에 의하면 염증보다는 섬유화가 두드러지며 미상의 원인물질 흡입에 의한 발생으로 추정되었다.

2011년 5월 18일

2011년의 원인 미상 폐질환과 2006년 이후 급성 간질성 폐렴(acute interstitial pneumonitis, AIP)으로 보고된 소아 폐질환은 유사한 양상을 보이는 새로운 질병으로 판단하였다. 다만, 질병이 소강상태(역학조사 후 새로 발생한 환자가 없었다.)이고 시급한 원인 규명이 필요하다는 점에서 성인 역학조사를 수행한 후 소아 대상의 연구를 수행하기로 하였다. 원인 미상 폐질환에 대한 실행적 정의(working definition)에 따라 흉부 영상 판독 소견을 검토하여 잠재 환자를 확인하고, 임상 팀이 의무기록을 검토하여 최종 질환 여부를 판정하였다.

2011년 5월 19일

전국 규모의 역학조사 계획을 결정하였다. 첫째 호흡부전연구회, 흉부영상연구회, 소아알레르기호흡기질환 연구자 네트워크 등을 활용하여 (본 질환의 실행적 정의에 따른) 잠재적 환자를 파악한다. 둘째 잠재 환자의 영상 소견을 중앙 검토하여, 환자를 확인한다. 셋째 환자군의 정보를 종합하여 질병의 자연사 등을 파악한다. 넷째 대조군을 선정하여 설문조사하고 그 결과를 환자군과 비교함으로써 위험요인을 파악한다. 한편 (A병원 임상 팀이 위험요인으로 제기한) 가습기 살균제의 시료 분석을 시행하기로 결정하였다.

2011년 5월 24일

A병원의 경우 전자의무기록에 영상의학 소견 등의 검색 키워드를 적용하여 2006년 이후 A병원에서 발생한 환자를 파악하였다. 한편 현재 A병원에서 2011년에 발생한 환자는 11명이었다.

2011년 5월 25일

유행의 원인으로 조사하여야 할 설문조사 항목을 논의하였으며, 환자 및 동거 가족에게 조사하기로 하였다. 가습기 살균제는 계절상품으로 5월 이후 판매되지 않음을 확인하였다.

2011년 5월 26일

A병원 연구윤리위원회에서 환자군 조사연구에 대한 심의면제를 결정하였다. 질병 위험요인을 파악하기 위한 설문조사 증례기록지를 작성하였다. 수거된 가습기 살균제 3가지는 관련 기관에 검사를 의뢰하였고, 흡입독성을 포함한 동물실험 가능성을 문의하였다.

2011년 5월 27일

질병 위험요인을 파악하기 위한 역학 팀의 대면 면접조사가 처음 실시되었다.

2011년 5월 30일

호흡기내과의 호흡기 질환 환자, 산과의 출산 여성을 대조군으로 선정하기로 결정하였다.

2011년 6월 1일

질본 2차 자문회의에서 연구현황을 보고하였다. A병원 이외 환자에 대한 연구는 수행하기 어려운 상황이었다.

2011년 6월 2일

A병원에서 2011년 이전에 발생한 환자를 파악함으로써, 2011년에 유행한 원인 미상 폐질환은 2011년 이전에 이미 발생한 질환임을 확인하였다. 가습기 살균제의 화학 성분을 확인하고, 동물실험을 실시할 방안을 검토하였다.

2011년 6월 3일

A병원에서 확인된 환자 30여 명(추정치)과 대조군 120여 명에 대해 위험요인을 파악하기 위한 설문조사를 수행하기로 결정하였다. 대조군은 폐질환 환자 1명당 A병원에 내원한 환자 중 4명을 성별, 나이, 지역, 입원 시기 등에 맞추어 선정하기로 결정하였다.

2011년 6월 7일

환자-대조군 연구는 A병원에서 시작하고 이후 다른 병원으로 확대할 것으로 정하였으며, 가습기 살균제에 대한 동물흡입 독성시험의 가능성을 검토하였다.

2011년 6월 8일

2011년 이전에 발생한 것으로 확인된 A병원 환자를 대상으로 전화 설문을 시작하였다. 설문조사 중 상당수 환자에서 거주 환경에서 곰팡이에 노출된 경우가

상당수 관찰되어 진균 감염 가능성을 검토하였다.

2011년 6월 10일

환자 가정을 방문하여 거주지 환경을 측정하였다. 가습기 살균제 제품의 각종 제품정보(MSDS 등)를 파악하였다.

2011년 6월 13일

환자 전원을 가정방문하여 심층 면접조사하기로 결정하였다.

2011년 6월 14일

A병원에서 발생한 환자를 실행적 정의에 따라 확인한 결과 2001년 이후 총 32명이었다(의심 환자 일부 포함).

2011년 6월 17일

가습기 살균제에 대한 동물흡입 독성시험을 수행할 기관을 결정하였다.

2011년 6월 22일

A병원 연구윤리위원회에서 대조군 설문조사 연구를 승인하여, 대조군 조사가

시작되었다. 가습기 살균제 중 세퓨 제품에는 PGH(Oligo(2-(2-ethoxy)ethoxyethyl guanidium chloride) 성분, 옥시, 롯데마트, 홈플러스 제품에는 PHMG(polyhexamethylene guanidine) 성분이 포함된 것을 확인하였다.

2011년 6월 23일

원인 미상 중증 폐질환 연구 진행보고회를 개최하였다. A병원에서 확인된 환자는 2011년 18명, 2006년부터 2010년까지 11명이었다. 2001년, 2004년, 2005년에도 각각 1명씩 3명의 환자가 발생하였다. 따라서 A병원에서 확인된 환자는 총 32명이었다. 이후 의심 환자 중 일부가 배제되어 총 28명으로 확인되었다. 타 병원에서 발생한 환자는 2006년부터 2010년까지 10명, 2011년 18명으로 추정되었다. A병원에서 설문조사가 완료된 인원은 2010년까지 7명, 2011년 11명, 총 18명이었다. 여자 15명 중 14명이 가습기 살균제를 사용하였고, 남자 3명은 전원이 사용하였다. 대조군은 여성 환자 1명당 호흡기 질환자 2명, 산모 2명, 남자 환자는 호흡기 질환이 있는 4명으로 선정하기로 결정하였다.

2011년 6월 24일

대조군 설문 항목을 최종 검토하여 설문조사 양식지를 확정하였다.

2011년 6월 27일

6월 23일 환자를 임상적으로 재검토한 결과 6월 27일 현재 A병원에서 발생한 환자는 2011년 18명, 2011년 이전 환자가 11명, 총 28명이었다.

2011년 6월 28일

“원인 미상 폐 손상” 자문회의를 개최하였다.

2011년 6월 29일

6월 27일부터 수행한 대조군의 대면 설문조사가 완료되었다.

2011년 6월 30일

호흡기 바이러스 검사결과가 최종 확인되었는데, 질병의 원인으로 의심되는 소견이 없었다. 대조군에 알레르기 내과 환자를 추가하여 조사하였다.

2011년 7월 1일

역학조사의 일환으로 가습기 살균제 성분조사나 동물실험을 병행하기로 결정하였다.

2011년 7월 4일

가족 집적성이 있는 가정을 방문조사하고 주거 환경 및 환경 검체를 채취하였다.

2011년 7월 7일

잠정 분석결과 가습기 살균제 사용 경험에 따른 질병 교차비 63배, 가습기 16배, 곰팡이 6배 등으로 추정되었다.

2011년 7월 8일

가습기 살균제에 대한 동물 흡입독성 시험 수행기관을 안전성 평가 연구소로 결정하였다. 기존에 확인한 29명 중 한 명은 다른 질환의 가능성이 더욱 높은 것으로 판단하여 최종적으로 A병원 환자군은 28명으로 확정되었다.

2011년 7월 11일

추가로 22명의 대조군 설문조사를 실시하였다. 외부 병원 환자 조사는 질본 공문 발송 후에도 실행 가능성이 낮을 것으로 생각되고, A병원 환자-대조군 연구 결과로 충분한 역학적 증거를 얻은 것으로 판단하여, 환자-대조군 연구는 최종 종료하였다.

2011년 7월 12일

질본 및 보건복지부에 연구결과를 보고하였다. 현재 가습기 살균제 노출, 가습기, 곰팡이 노출의 교차비는 각각 54.4, 15.5, 4.5이었다. 성별, 연령, 곰팡이 노출 등을 보정한 다변량 분석결과도 동일한 양상이었다. 이후 산과 환자를 대조군으로 추가하여 대조군을 확정한 후 최종 교차비를 계산할 것이다. 가습기 살균제는 역학적으로는 원인이라고 간주되었다. 이후 동물실험, 특히 흡입독성 시험이 필요하며 가습기 살균제의 성분을 정확히 확인할 필요가 있었다.

2011년 7월 13일

타 병원 환자군 조사나 대조군 선정은 진행하지 않는 것으로 최종 확정하였고, 지역사회 대조군 조사만 추가로 실시하기로 하였다. A병원 환자 대조군 연구는 현재까지 분석한 결과를 최종 분석결과로 확정하기로 결정하였다. 동물실험 등의 생물학적 증거를 보완할 필요가 있다고 논의하였다.

2011년 7월 18일

보건복지부, 질본, 역학전문위원회에 연구결과를 최종 보고하였다. A병원에서 원인 미상 폐 손상으로 확정된 환자는 28명(2011년 17명, 이전 11명), 설문 조사

된 환자는 18명(2011년 11명, 이전 7명)이었다. 대조군은 121명으로 확정하였다. 단변수 분석결과 가습기 살균제 노출에 대한 교차비는 47.3, 가습기를 사용할 때 13.7배, 집에서 곰팡이가 관찰된 경우 위험도가 4.4배 높았다. 곰팡이 노출, 성별, 연령을 보정한 다변량 분석결과 가습기 살균제의 교차비는 48.8이었다. 이후 가습기 살균제에 대한 폐 세포 독성시험, 동물 흡입 독성시험을 실시할 것을 결정하였다.

2011년 7월 20일

8월부터 소아 환자에 대한 연구를 시작하기로 결정되었다. 가습기 살균제에 대한 환자군 심층조사 및 가정방문조사는 7월 21일부터 시작기로 하고 심층면담 계획을 확정하였다.

2011년 7월 21일

인과성을 명확히 규명하여야 할 현실적 필요성 등을 감안하여 동물실험 자료, 역학 연구에서도 양-반응 관계, 질병 자연사에 대한 검토를 통해 추가 증거를 확보하는 것이 바람직하다고 (전문가 자문 및 연구진 논의를 거쳐) 판단하였다. 환자군 가정방문 심층면접 조사를 시작하였다.

2011년 7월 25일

기존 설문지에 가습기 살균제 양-반응 관계 등을 평가하는 문항을 추가하여 지역사회 대조군 설문조사를 시행하기로 결정하였다. 소아 대상 역학연구, 세포 및 동물실험 연구는 질본에서 총괄하여 진행하기로 결정하였다. 소아 대상의 역학연구에는 A병원 역학 팀이 연구진으로 참여하여 유사한 연구설계로 진행되었다.

2011년 8월 1일

환자군 가정방문 심층면접 조사를 위한 심층설문지와 지역사회 대조군 설문지를 최종 확정하였다. 역학적 인과성을 규명하기 위한 생물학적 증거로서 세포독성 시험과 기도 내 점적(instillation) 동물시험을 진행하여 8월내 조사결과를 발표하기로 결정하였다.

2011년 8월 8일

환자군 심층조사 및 가정방문조사가 7월 21일부터 시행하여 총 15명을 조사하였다. 동물흡입독성 시험의 전 단계 연구로서 (공기 중 배출되는 입자의 크기 등을 파악하기 위한) 가습기 살균제 발생 시험을 진행하였다.

2011년 8월 11일

세포독성 시험의 중간결과가 보고되었다. 50% 치사농도(LC50)를 계산하였으며 폐 세포 사멸 효과가 관찰되었다.

2011년 8월 18일

가습기 살균제 발생시험 결과가 보고되었다. 말단 세기관지까지 투과 가능할 정도의 미세한 가습기 살균제 입자가 분출되는 것을 확인하였다.

2011년 8월 30일

역학연구결과 및 세포 실험, 입자 배출시험 결과 등을 “원인 미상 폐 손상” 관련 전문가 자문회의(역학조사 전문위원회 위원, 감염병, 환경보건, 독성, 호흡기 내과 등 자문위원 및 용역 연구진, 질본 내부 관계관 참석)에서 보고하고 토론하였다. 안전성 평가 연구소의 입자 배출시험 결과 세기관지, 폐 말단까지 가습기 살균제가 투과 가능한 것을 확인하였고, 세포독성시험으로 가습기 살균제의 세포 독성을 확인하였다. 또한 기도 내 점적 방식으로 가습기 살균제를 노출한 동물실험에서 체중 감소, 조직병리학적 염증 소견 등이 관찰되어 동물에 대한 독성이 있음을 확인하였다.

2011년 8월 31일

역학조사 결과 및 세포독성 시험을 토대로 기자회견을 개최하여 가습기 살균제가 원인 미상 폐 손상의 위험요인으로 추정되므로, 최종 인과관계가 확인될 때까지 사용 및 출시를 자제할 것을 권고하였다.

2011년 9월 2일

소아 대상 연구를 위한 사전 모임을 개최하였다.

2011년 9월 22일

원인 미상 중증 폐 질환에 대한 연구용역 착수회의를 개최하였다. 본 과제는 2011년 전국적으로 발생한 원인 미상 중증 폐질환의 발생 건수를 파악하기 위한 목적으로 수행되었다.

2011년 11월 10일

원인 미상 폐 손상 역학조사와 관련하여 2차 전문가 자문회의를 개최하였다. 동물흡입독성 시험의 진행사항을 확인하고, 부검 후 폐의 조직학적 소견을 검토하였다. 옥시 제품과 세퓨에 노출된 쥐의 병리학적 소견은 원인 미상 폐질환 환자의 소견과 동일한 것으로 확인되었다.

2011년 11월 11일

보건복지부에서 원인 미상 폐 손상의 원인이 가습기 살균제인 것으로 발표하였으며 살균제 6종의 강제 수거 명령이 내려졌다.

2012년부터 현재까지

가습기 살균제로 인해 발생한 성인 및 소아의 폐 손상 사례가 보고되지 않았다. 또한 본 환자-대조군 연구 방식으로 수행한 소아 간질성 폐렴 대상의 환자-대조군 연구에서도 가습기 살균제가 원인으로 밝혀졌다.

III. 가습기 살균제 역학조사 이후

가습기 살균제로 인한 폐 손상의 역학조사는 새로운 질병을 확인하고 그 인과적 원인을 규명한 드문 사례이다. 본 역학조사의 특징과 한계, 교훈 등을 몇 가지 언급하고자 한다.

첫째 가습기 살균제 역학조사는 일개 병원에서 신고한 질병 유행(epidemic)에 대해 그 병원과 의과대학 연구진(호흡기내과, 영상의학과, 병리학과, 소아과, 예방

의학 20명 이상), 질병관리본부 역학조사관과 지휘부, 외부 대학과 연구소의 전문가 연인원 20명 이상이 협력하여 다학제 연구로 진행하였다.

둘째 전국에서 발생한 유행의 원인을 일개 병원 자료만으로 규명하였다. 새로운 질병의 유행이라는 임상적 컨센서스가 없는 상태, 개인정보보호법에 의해 시의적절한 의료정보 수집이 어려운 점, 공익 목적 연구의 경우에도 신속한 연구윤리심의 절차가 부재한 점 등의 이유로 단일 기관 역학조사를 수행하는 것이 불가피하였다.

셋째 새로운 질병 여부를 판단하고, 유행 규모를 추정하고, 질병의 실행적 정의를 내리는데 전자의무기록을 적극적으로 활용하였다. 본 역학조사에서는 초기에 보고된 환자들의 임상, 영상의학, 병리 소견을 요약 정리하는 과정에서 간유리음영 등 공통 키워드를 도출하였다. 도출된 키워드를 병원의 전자의무기록에 적용하여 빠른 시간에 잠재적 환자를 선별하고 이를 의료진과 영상의학 전문가가 최종 판정함으로써 환자를 확인하였다.

넷째 역학연구로써 가습기 살균제가 위험요인으로 확인된 후 그 생물학적 개연성을 증명하기 위한 실험연구가 신속히 진행되었다. 가습기 살균제는 기업에서

대규모로 다수의 사람에게 판매하는 제조물이기 때문에 역학연구에서 제시하는 교차비의 크기, 양-반응 관계 정도의 역학적 증거 외에도 생물학적 개연성을 보여주는 실험적 증거가 필요하다. 동물흡입 독성시험의 노출 환경, 살균제 농도 등을 결정하는데 독성학은 물론, 역학, 임상 연구진이 참여하여 합리적인 계획을 수립할 수 있었으며, 성공적으로 독성의 객관적 증거를 도출할 수 있었다.

다섯째 역학조사는 임상적 지식을 토대로 역학적 기법을 적용하여 수행하여야 한다. 따라서 임상의사와 역학자는 역학조사의 모든 의사결정과 연구수행 과정에 긴밀히 협력하는 것이 필수적이다. 긴밀한 의사교환 없이는 두 가지 다른 자료, 결과, 최악의 경우 두 가지 다른 결론이 얻어질 수 있기 때문이다. 역설적으로 (다기관 연구가 불가능하여) A병원의 임상의사와 역학자가 A병원 자료만으로 조사를 수행한 점이 본 연구의 원활하고 성공적인 수행을 가능하게 한 것으로 생각된다.

(감사의 글) 2011년 발생한 산모 중증 호흡기질환의 역학조사에서 질병관리본부는 역학조사 착수를 신속히 결정하고, 연구진으로 직접 참여하여 가습기 살균제가 원인임을 성공적으로 밝혀냈다. 역학조사 수행을 결정하고 이를 지원한 질병관리본부 본부장, 감염병센터장에게 감사하고 싶다.

IV. 참고문헌

가습기 살균제 중증 폐질환의 역학조사와 관련하여 보고된 논문을 아래와 같이 정리하였다.

1. Park JH, Kim HJ, Kwon GY, Gwack J, Park YJ, Youn SK, Kwon JW, Yang BG, Lee MS, Jung M, Lee H, Jun BY, Lim HS. Humidifier Disinfectants Are a Cause of Lung Injury among Adults in South Korea: A Community-Based Case-Control Study. PLoS One. 2016 Mar 18;11(3):e0151849.

2. Kim HJ, Lee MS, Hong SB, Huh JW, Do KH, Jang SJ, Lim CM, Chae EJ, Lee H, Jung M, Park YJ, Park JH, Kwon GY, Gwack J, Youn SK, Kwon JW, Yang BG, Jun BY, Kim Y, Cheong HK, Chun BC, Kim H, Lee K, Koh Y. A cluster of lung injury cases associated with home humidifier use: an epidemiological investigation. Thorax. 2014 Aug;69(8):703-8.

3. Hong SB, Kim HJ, Huh JW, Do KH, Jang SJ, Song JS, Choi SJ, Heo Y, Kim YB, Lim CM, Chae EJ, Lee H, Jung M, Lee K, Lee MS, Koh Y; Korean Unknown Severe

Respiratory Failure Collaborative; Korean Study Group of Respiratory Failure. A cluster of lung injury associated with home humidifier use: clinical, radiological and pathological description of a new syndrome. Thorax. 2014 Aug;69(8):694-702.

4. Kim KW, Ahn K, Yang HJ, Lee S, Park JD, Kim WK, Kim JT, Kim HH, Rha YH, Park YM, Sohn MH, Oh JW, Lee HR, Lim DH, Choung JT, Han MY, Lee E, Kim HY, Seo JH, Kim BJ, Cho YA, Do KH, Kim SA, Jang SJ, Lee MS, Kim HJ, Kwon GY, Park JH, Gwack J, Youn SK, Kwon JW, Jun BY, Pyun BY, Hong SJ. Humidifier disinfectant-associated children's interstitial lung disease. Am J Respir Crit Care Med. 2014 Jan 1;189(1):48-56.

5. Yang HJ, Kim HJ, Yu J, Lee E, Jung YH, Kim HY, Seo JH, Kwon GY, Park JH, Gwack J, Youn SK, Kwon JW, Jun BY, Kim KW, Ahn K, Lee SY, Park JD, Kwon JW, Kim BJ, Lee MS, Do KH, Jang SJ, Pyun BY, Hong SJ. Inhalation toxicity of humidifier disinfectants as a risk factor of children's interstitial lung disease in Korea: a case-control study. PLoS One. 2013 Jun 5;8(6):e64430.
